# Krüppel-like Factor 2 (KLF2) in the Regulation of Lipid Accumulation, ROS, and Mitochondrial Functions During Foam Cell Formation in RAW264.7 Cells

**DOI:** 10.3390/biology15020111

**Published:** 2026-01-06

**Authors:** Md Sariful Islam Howlader, Manjusri Das, Surajit Hansda, Prathyusha Naidu, Hiranmoy Das

**Affiliations:** Department of Pharmaceutical Sciences, Jerry H. Hodge School of Pharmacy, Texas Tech University Health Sciences Center, Amarillo, TX 79106-1712, USA; mdhowlad@ttuhsc.edu (M.S.I.H.); mandas@ttuhsc.edu (M.D.); shansda@ttuhsc.edu (S.H.); pratnaid@ttuhsc.edu (P.N.)

**Keywords:** foam cell, KLF2, lipid uptake, reactive oxygen species, mitochondria

## Abstract

Foam cell formation, a hallmark of early atherosclerotic lesion development, is closely associated with mitochondrial dysfunction and excessive reactive oxygen species (ROS) production. Disruption in mitochondrial activity leads to electron leakage, elevated ROS generation, and collapse of mitochondrial membrane potential, contributing to vascular pathogenesis. In this study, we investigated the role of Krüppel-like factor 2 (KLF2), a transcription factor known for its vasculoprotective effects, in regulating mitochondrial function during foam cell (FC) formation in RAW264.7 cells. This study demonstrates that KLF2 is decreased during FC formation of RAW264.7 cells. In contrast, lipids are highly uptaken, and both intracellular and mitochondrial ROS are increased, with enhanced mitochondrial membrane potential and mitochondrial functions during FC formation of RAW264.7 cells. To investigate the role of KLF2 in this FC formation process, we utilized both loss-of-function and gain-of-function approaches of KLF2 in RAW264.7 cells. This study demonstrates that KLF2 plays a multifaceted and protective role in preventing FC formation by regulating the uptake of lipids, reducing both intracellular and mitochondrial ROS, mitochondrial membrane potential, and mitochondrial activities, as loss-of-function of KLF2 promoted FC formation with overactivity, and gain-of-function reduced FC formation by limiting activities of all the parameters mentioned above. These findings provide mechanistic insights into the protective role of KLF2 and propose it as a potential therapeutic target for the future management of cardiovascular diseases.

## Simple Summary

This study defines the role of KLF2, which decreases during foam cell (FC) formation of myeloid cells. We found that lipids are rapidly taken up, and both intracellular and mitochondrial ROS levels increased during FC formation. Mitochondria undergo depolarization and enter a state of dysfunctional hyperactivity. To examine KLF2′s role in this process, we employed both loss-of-function and gain-of-function approaches in RAW264.7 cells. The study demonstrates that KLF2 plays a multifaceted and protective role in preventing FC formation by regulating lipid uptake and reducing both intracellular and mitochondrial ROS, mitochondrial membrane potential, and mitochondrial activity. Loss of KLF2 results in increased FC formation with overactivity, while gain-of-function reduces FC formation by limiting these parameters. These findings provide mechanistic insights into the protective role of KLF2 and suggest it as a potential therapeutic target for future cardiovascular disease management.

## 1. Introduction

Atherosclerosis remains a leading cause of morbidity and mortality worldwide, caused by the persistent buildup of lipid-rich plaques in arterial walls. An early cellular event in this disease process is the formation of foam cells (FC) from monocytes that have taken up large amounts of oxidized low-density lipoproteins (ox-LDL). This lipid intake, primarily facilitated by scavenger receptors such as CD36 and LOX-1, leads to cholesterol accumulation, which triggers oxidative stress and inflammatory signals that drive plaque growth.

Mitochondria play a crucial role in foam cell formation by regulating cellular energy production and maintaining redox balance. In the mitochondrial electron transport chain (ETC), Complex I (NADH: ubiquinone oxidoreductase) and Complex III (cytochrome bc1 complex) are key components of oxidative phosphorylation (OXPHOS), supporting electron transfer and proton pumping to generate ATP. However, these complexes are also the main sources of mitochondrial reactive oxygen species (ROS), especially superoxide anion (O_2_•^−^), particularly when their function is impaired or overloaded [1,2]. Dysfunction in Complexes I or III can disrupt electron flow, leading to electron leakage and increased ROS production. This process then compromises the mitochondrial membrane potential, reduces ATP output, and triggers cell death pathways, all of which contribute to foam cell formation and the development of atherosclerotic plaque [3,4].

Experimental studies have shown that pharmacologic targeting of these mitochondrial complexes can alter disease outcomes. Rotenone, a classical Complex I inhibitor, while toxic at high doses, has paradoxically been found to reduce oxidative stress and vascular smooth muscle cell (VSMC) proliferation in certain disease models [5,6]. Likewise, azoxystrobin, an inhibitor of Complex III, can attenuate VSMC activation in response to ox-LDL and has shown partial effectiveness in reducing foam cell formation [7]. These studies highlight the potential benefits of adjusting mitochondrial activity for the development of a therapeutic strategy for vascular diseases.

The production of ROS by Complex I and III is essential in controlling mitochondrial health and cellular signaling. Under normal conditions, electrons flow through the ETC efficiently, reducing oxygen to water. However, during stress or disease states, electron leakage can happen, especially in Complexes I and III, leading to increased ROS production. While Complex I typically releases superoxide into the mitochondrial matrix, Complex III can do so in both the matrix and the intermembrane space [1,6,7,8]. This ROS overload damages mitochondrial DNA, proteins, and lipids, impairing the structural integrity of the mitochondria and lowering mitochondrial membrane potential. The resulting dysfunction triggers apoptosis and promotes inflammatory responses that contribute to foam cell formation and plaque vulnerability [2,3].

Krüppel-like factor 2 (KLF2) has emerged as a critical regulator of vascular homeostasis and inflammation [9,10,11,12,13,14]. Originally characterized in endothelial cells for its role in mediating shear stress responses, KLF2 is now known to be expressed in RAW264.7 cells, where it exerts a broad range of protective effects. It limits inflammatory expression, enhances cholesterol efflux, and activates antioxidant pathways. Importantly, KLF2 appears to preserve mitochondrial function by stabilizing mitochondrial membrane potential, reducing mitochondrial ROS, and sustaining oxidative metabolism [15,16]. In RAW264.7 cells exposed to ox-LDL, KLF2 expression is often suppressed, leading to mitochondrial dysfunction and accelerated foam cell formation.

The activation of KLF2 using a chemical compound, GGTI298 (an inhibitor of geranylgeranyltransferase I) has shown encouraging results in restoring mitochondrial membrane potential and lowering ROS levels [9,12,17]. The fact that similar protective effects are observed with GGTI298 suggests that KLF2 may act, at least in part, by modulating the function of mitochondrial Complexes I and III. This suggests a broader role for KLF2 in regulating mitochondrial superoxide production and bioenergetic stability.

Given the pivotal contribution of mitochondrial dysfunction and oxidative stress to FC development, this study was designed to define the role of KLF2 in modulating these pathological processes in monocytes, as monocytes give rise to FCs after uptaking ox-LDL. Using a monocytic cell line, RAW264.7 cells as a model system, we examined the impact of KLF2 on cellular and mitochondrial ROS generation, mitochondrial membrane potential, and metabolic function. To dissect the regulatory role of KLF2, we employed a chemical, GGTI298, to enhance KLF2 expression and a molecule, GGPP, to suppress expression of KLF2, allowing us to evaluate how shifts in KLF2 activity influence mitochondrial health, function, and lipid accumulation during monocyte differentiation toward FCs. By integrating confocal microscopy, immunocytochemical staining, and Seahorse-based metabolic profiling, our findings highlight KLF2 as a key modulator for maintaining mitochondrial activity towards a healthy condition. Ultimately, this work provides new insight into the mechanisms by which KLF2 may counteract the initiation of FC formation, underscoring its potential as a therapeutic target in atherosclerotic disease.

## 2. Materials and Methods

### 2.1. Reagents and Materials

The RAW264.7 cells were obtained from the American Type Culture Collection (ATCC, Manassas, VA, USA, #TIB-71). Oxidized low-density lipoprotein (ox-LDL) (Ann Arbor, MI, USA, #601,181), geranylgeranyl pyrophosphate (GGPP, Ann Arbor, MI, USA, CAS #313263-08-0), and GGTI298 (Ann Arbor, MI, USA, CAS #1217457-86-7) were purchased from Cayman Chemical (Ann Arbor, MI, USA). The fluorescent probe 2′,7′-dichlorodihydrofluorescein diacetate (DCFDA, #4091-99-0) was obtained from Sigma-Aldrich (St. Louis, MO, USA). 4′,6-diamidino-2-phenylindole dihydrochloride (DAPI, #D1306) and TRIzol™ Reagent (#15596026) were procured from Invitrogen (Carlsbad, CA, USA). The High-Capacity RNA-to-cDNA Kit (#4387406) and SYBR™ Green PCR Master Mix (#4309155) were supplied by Applied Biosystems (Foster City, CA, USA). JC-1 Dye (#T3168), used for assessing mitochondrial membrane potential, and MitoSOX™ Red (#M36008), used for detecting mitochondrial superoxide, were purchased from Thermo Fisher Scientific (Waltham, MA, USA). A 4% paraformaldehyde (PFA) solution (#sc-281692) was obtained from Santa Cruz Biotechnology (Dallas, TX, USA). Oil-Red-O stain (catalog #MAK194), 10% neutral buffered formalin (catalog #252549), and isopropanol (CAS #67-63-0) were obtained from Sigma-Aldrich. The Seahorse XFp Cell Mito Stress Test Kit (catalog #103010-100) was purchased from Agilent Technologies.

### 2.2. Cell Culture and Treatment Conditions

RAW264.7 murine macrophage cells were maintained in Dulbecco’s Modified Eagle Medium (DMEM) supplemented with 10% fetal bovine serum (FBS) and 1% penicillin-streptomycin under standard conditions (37 °C, 5% CO_2_). Before stimulation, cells were serum-starved for 6 h to synchronize metabolic activity. For foam cell differentiation, cells were then incubated with oxidized low-density lipoprotein (Ox-LDL) at the optimized concentration, as determined in preliminary experiments. Where indicated, GGTI298 (20 μM) or GGPP, 10 μM was added at the start of the differentiation process and maintained throughout the culture period (six days) until termination of the experiment. This design ensured continuous modulation of KLF2 activity during foam cell formation. All treatments were performed in parallel with appropriate vehicle controls.

### 2.3. MTT Assay

Cells were seeded into 96-well plates at a density of 1 × 10^4^ cells/well and allowed to attach overnight. Treatments included: (i) untreated control, (ii) RAW264.7 cells treated with GGTI298 (0–100 µM), and (iii) RAW264.7 cells treated with GGPP (0–100 µM). Fresh media containing the appropriate treatments were replaced every 48 h. The duration of treatments was extended up to 6 days. Cell viability was assessed using the MTT [3-(4,5-dimethylthiazol-2-yl)-2,5-diphenyl tetrazolium bromide] assay (Sigma-Aldrich, St. Louis, MO, USA) as previously described [18,19]. At the end of the treatment period (days 1–6), 10 µL of MTT reagent (5 mg/mL) was added to each well and incubated for 4 h at 37 °C. Following incubation, the supernatant was aspirated, and the formazan crystals were dissolved in 100 µL of dimethyl sulfoxide (DMSO; Sigma-Aldrich, St. Louis, MO, USA). Absorbance was measured at 570 nm using a microplate reader (BioTek Synergy H1, Agilent Technologies, Santa Clara, CA, USA). Viability was expressed as a percentage of the untreated control (set as 100%). Experiments were performed in triplicate, and data are presented as mean ± standard deviation (SD).

### 2.4. Oil Red O Staining for Bright Field and Immunocytochemistry

Oil Red O staining was conducted to assess intracellular lipid accumulation in RAW264.7 cells under both bright field and immunofluorescence microscopes. After various experimental conditions, cells were rinsed gently with phosphate-buffered saline (PBS) and fixed with 10% (*v*/*v*) neutral buffered formalin for 15 min at room temperature. Following fixation, cells were washed three times with double-distilled water (ddH_2_O) and stained for 15 min with freshly prepared Oil Red O working solution, obtained by diluting a 0.5% Oil Red O stock solution (in isopropanol) with ddH_2_O at a 3:2 ratio (*v*/*v*), followed by filtration through a 0.45 μm membrane filter. Post-staining, cells were rinsed three times with ddH_2_O to remove background staining. For bright field analysis, stained lipid droplets were imaged under a standard light microscope at 100× magnification. For immunofluorescence, Oil Red O-stained cells were counterstained with DAPI and imaged using a Leica Stellaris 8 STED super-resolution confocal microscope, which is equipped with a 100× oil immersion objective. Fluorescent images were captured using a 510 nm excitation wavelength to detect the intrinsic red fluorescence of Oil Red O, and mean fluorescence intensity (MFI) was quantified using LAS X image analysis software (https://www.leica-microsystems.com/products/microscope-software/p/leica-las-x-ls/, accessed on 11 August 2025, Leica Microsystems, Wetzlar, Germany). Oil-Red-O staining was performed after 6 days of ox-LDL treatment to evaluate intracellular lipid accumulation associated with foam cell formation. For quantitative lipid analysis, Oil Red O dye retained within the cells was eluted using 100% isopropanol, and absorbance was measured at 510 nm with a spectrophotometer.

### 2.5. Evaluation of Mitochondrial Membrane Potential

Mitochondrial membrane potential was assessed in cells after staining with JC-1 dye. In brief, RAW264.7 cells (1 × 10^4^ cells/well) were cultured on coverslips for 6 days in the presence of FC differentiation medium, with or without GGTI298 (20 μM) or GGPP (10 μM) in the cell culture. Cells were then rinsed with ice-cold PBS and stained with JC-1 dye (Thermo Fisher Scientific) at 37 °C for 20 min. After thoroughly washing with PBS, cells were mounted with DAPI and imaged using the Leica Stellaris 8 STED super-resolution confocal microscope (100× objective) (Wetzlar, Germany). JC-1 staining was conducted after 6 days of treatment to assess alterations in mitochondrial membrane potential during foam cell differentiation. Image quantification of green (monomeric JC-1) and red (aggregated JC-1) fluorescence was performed using LAS X software (Leica Microsystems, Wetzlar, Germany) to assess mitochondrial polarization. Three biological replicates were analyzed, with five fields imaged per coverslip.

### 2.6. Assessment of Intracellular Reactive Oxygen Species

Intracellular reactive oxygen species (ROS) were evaluated after staining cells with the DCFDA (2′,7′-dichlorofluorescin diacetate) dye. In brief, RAW264.7 cells were seeded at a density of 1 × 10^4^ cells/well on sterile glass coverslips and cultured for 6 days in foam cell (FC) differentiation media in the presence or absence of the optimized concentrations of GGTI298 (20 μM) or GGPP (10 μM). Cells were then washed with PBS and incubated with 20 μM DCFDA dye at 37 °C for 30 min. After incubation, coverslips were rinsed thoroughly with PBS and mounted on glass slides using a mounting medium containing DAPI. Fluorescence images were captured using a Leica Stellaris 8 STED super-resolution (Leica Microsystems, Wetzlar, Germany) confocal microscope equipped with a 100× oil immersion objective. Quantification of ROS levels was performed using LAS X image analysis software. Three independent experiments were conducted, and five random fields per coverslip were imaged for quantification.

### 2.7. Detection of Mitochondrial Superoxide Production

Mitochondrial superoxide levels were measured in cells after staining with MitoSOX™ Red. In brief, RAW264.7 cells (1 × 10^4^ cells/well) were cultured on coverslips under the same differentiation conditions as described above. After 6 days, cells were washed with ice-cold PBS and incubated with a 2 μM concentration of MitoSOX Red at 37 °C for 30 min. Following staining, cells were washed with PBS and mounted on the slide using DAPI. Fluorescent images were acquired using the Leica Stellaris 8 STED confocal microscope with a 100× objective, and image analysis was performed using LAS X software (Leica Microsystems, Wetzlar, Germany). Three independent experiments were performed, and five random fields per sample were analyzed for quantification.

### 2.8. Mitochondrial Respiration Analysis

To investigate mitochondrial respiratory function, the Seahorse XF Cell Mito Stress Test was performed using an Agilent Seahorse XFe24 extracellular flux analyzer (New York, NY, USA). In brief, RAW264.7 cells (1 × 10^4^ cells/well) were seeded on Seahorse XF24 cell culture microplates and differentiated for 6 days in the presence or absence of GGTI298 (20 μM) or GGPP (10 μM). On the day of the assay, culture medium was replaced with Seahorse XF assay medium (Agilent Technologies) supplemented with 1 mM pyruvate, 2 mM glutamine, and 10 mM glucose, and the cells were incubated at 37 °C in a non-CO_2_ containing incubator for 1 h. Oxygen consumption rate (OCR) was measured following sequential addition of 1.5 μM oligomycin (ATP synthase inhibitor), 1 μM FCCP (uncoupler), and 2 μM rotenone/antimycin A (complex I/III inhibitors). Data was acquired and analyzed using Seahorse Wave software version 2.6.1 (New York, NY, USA). Four independent replicates were used for each treatment condition, and mitochondrial respiration parameters, including basal respiration, ATP production, maximal respiration, and spare respiratory capacity, were calculated. ECAR was not assessed, as it is not relevant to the mitochondrial pathway being investigated.

### 2.9. Statistical Analysis

Each experiment was performed at least three times, and each data point was measured in triplicate. Data are presented as mean ± SEM from three independent experiments. Statistical significance was determined using one-way ANOVA followed by Tukey’s post hoc test, with *p* < 0.05 considered significant.

## 3. Results

### 3.1. Effect on the Expression of KLF2 During Foam Cell Formation

The MTT assay revealed that RAW264.7 cells retained high viability when exposed to GGTI298 and GGPP across the tested concentrations (0–100 µM) for up to 6 days. In the untreated control group, cell viability remained stable at ~100% throughout the 6 days. Treatment with GGTI298 showed minimal cytotoxicity, with cell viability maintained above 90% even at 100 µM. In contrast, GGPP treatment produced a modest but consistent decline in viability, with ~85% viability observed at the highest concentration (100 µM) by day 6. These findings indicate that RAW264.7 macrophages are safe up to 100 µM for 6 days, with GGTI298-treated cells exhibiting higher metabolic activity compared to GGPP-treated cells.

**Figure 1:**
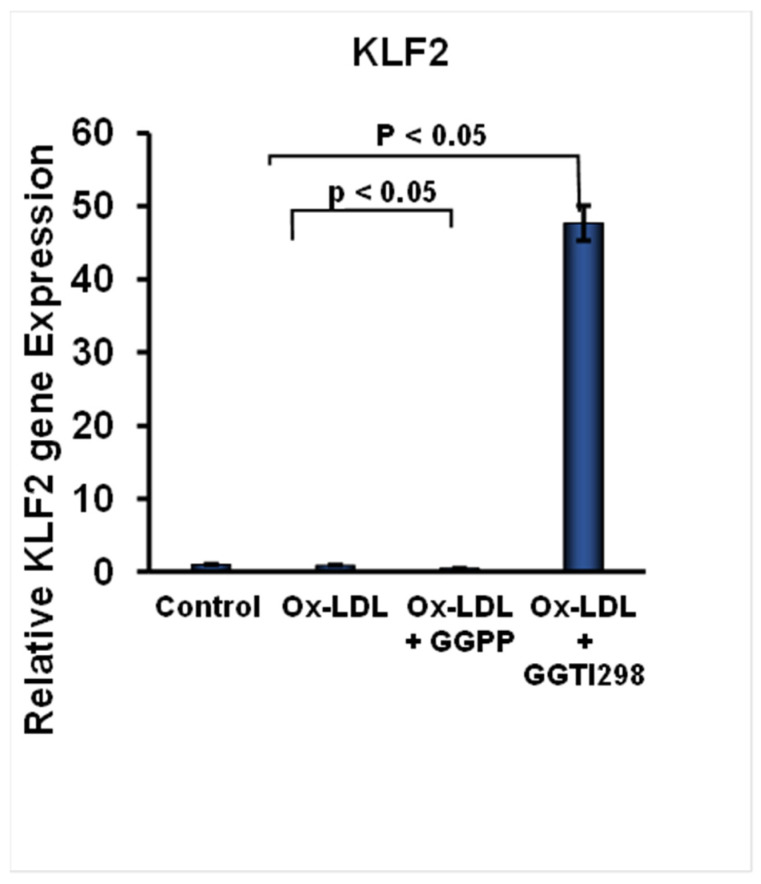
KLF2 expression is altered during foam cell formation and with compounds. KLF2 expression data are presented graphically after the quantitative reverse transcriptase polymerase chain reaction (qRT-PCR) analysis of cells during foam cell formation with ox-LDL in the presence of chemical compounds like GGPP or GGTI298. Each experiment was performed at least three times, and each data point was in triplicate. Data represent mean ± SEM from three independent experiments. Statistical significance was determined using one-way ANOVA followed by Tukey’s post hoc test.

The accumulation of lipid by monocytes is a critical early event for the development of foam cells and was effectively induced after the addition of ox-LDL to the culture medium. To investigate the expression of our target molecule KLF2, the qRT-PCR method was applied, results showing a significant decrease (*p* < 0.01) in the KLF2 expression after the formation of FC, which are depicted in Figure 1. This suppression of KLF2 expression was further intensified upon the addition of GGPP, a KLF2 inhibitor, resulting in an even more pronounced decrease in KLF2 expression (*p* < 0.01), as shown in Figure 1. These findings suggest that the presence of GGPP results in a substantial reduction in KLF2 expression. The loss of KLF2 might contribute to enhanced FC formation and the development of pathogenesis associated with foam cells (Figure 1).

To confirm the role of KLF2 in this process, we have added the GGTI298 to the RAW264.7 cells during the induction of FCs in the culture. There was a significant induction of KLF2 expression above its basal levels (*p* < 0.01) during the FC formation of RAW264.7 cells, Figure 1. These results demonstrate that chemical activation of KLF2 is effective in counteracting the suppressive effects of KLF2 expression during FC formation. These results suggest that there might be a protective role of KLF2 in maintaining RAW264.7 cells’ homeostasis.

### 3.2. Effect of KLF2 Modulation on Lipid Uptake by Monocytes

**Figure 2:**
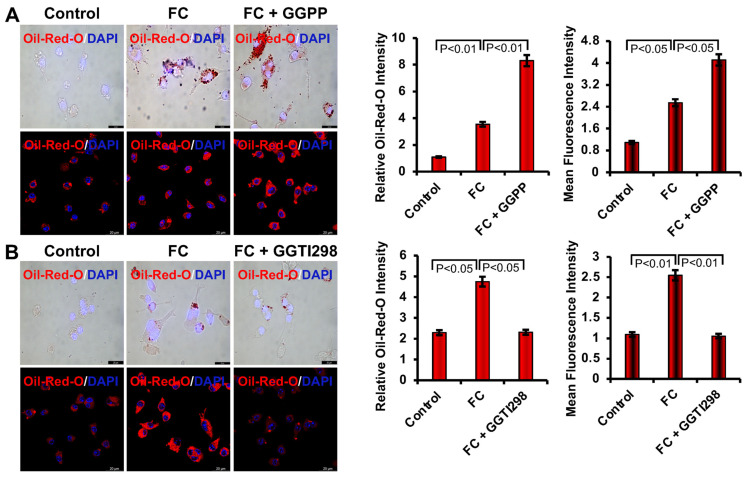
Inhibition of KLF2 increased the lipid uptake by monocytes, and overexpression of KLF2 decreased the uptake of lipids. (**A**). Bright field confocal microscopy images presented after the inhibition of KLF2 (after the addition of GGPP) during foam cell formation with oxidized low-density lipoprotein (ox-LDL) of RAW264.7 cells after Oil Red O staining and corresponding fluorescence images. Quantification of bright-field and fluorescence images is presented. (**B**). Similarly, bright field confocal microscopy images showing after the overexpression of KLF2 (after the addition of GGTI298) during foam cell formation with oxidized low-density lipoprotein (ox-LDL) of RAW 264.7 cells after Oil Red O staining and corresponding fluorescence images. Quantitative analysis of bright-field images and fluorescence images is shown. Each experiment was performed at least three times, and each slide was captured in at least five different areas. For graphical data, represent mean ± SEM from three independent experiments. Statistical significance was determined using one-way ANOVA followed by Tukey’s post hoc test. Scale bar = 20 µm.

After the addition of ox-LDL to the RAW264.7 cells, foam cell formation was visualized using light microscopy. This uptake of ox-LDL was characterized by a prominent accumulation of cytoplasmic lipid droplets and determined by intensified Oil Red O staining. However, after the addition of GGPP, a chemical inhibitor of KLF2 activity, in the presence of ox-LDL, the formation of FCs was significantly enhanced. This was demonstrated by an increased intensity of red lipid staining in bright field microscopy and a corresponding rise in fluorescence emission at 515 nm, indicating elevated intracellular lipid accumulation. In contrast, the addition of GGTI298, a chemical inducer of KLF2 activity and function, led to a substantial reduction in the formation of FCs. This was marked by a significant reduction in Oil Red O staining and a lower fluorescence signal, supporting the role of KLF2 in regulating lipid accumulation and limiting the formation of FCs by uptaking ox-LDL. Collectively, these findings underscore the regulatory role of KLF2 in RAW264.7 cells’ lipid homeostasis and its potential of being a target molecule in the development of FC (Figure 2).

### 3.3. Effect of KLF2 on the Transmembrane Potential of Mitochondria During FC Formation

To investigate the effect of KLF2 on the transmembrane potential of mitochondria during FC formation, we assessed mitochondrial membrane potential using JC-1 staining, a well-established indicator of mitochondrial polarization. JC-1 dye forms red-fluorescent aggregates in mitochondria with high membrane potential, while in depolarized mitochondria, it exists as green-fluorescent monomers [20].

**Figure 3:**
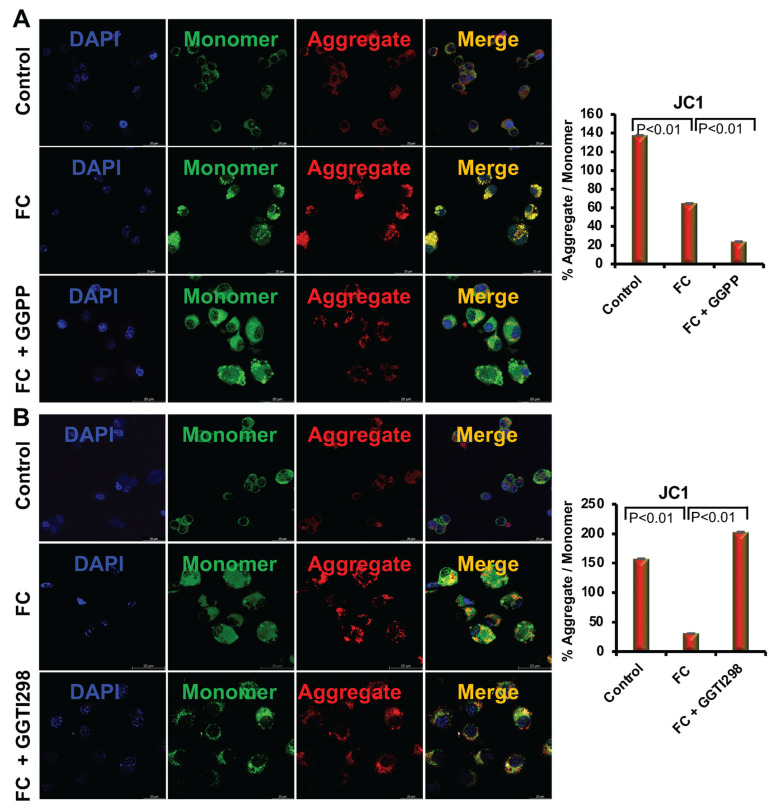
Inhibition of KLF2 disrupted the transmembrane potential of mitochondria, and overexpression of KLF2 restored it. (**A**). Representative confocal microscopy images were shown after the inhibition of KLF2 (after the addition of GGPP) during foam cell formation with oxidized low-density lipoprotein (ox-LDL) of RAW 264.7 cells after JC-1 staining (left upper panels). The upper right panel graphically shows the quantified data. (**B**). Representative confocal microscopy images were shown after the overexpression of KLF2 (after the addition of GGTI298) during foam cell formation with oxidized low-density lipoprotein (ox-LDL) of RAW 264.7 cells after JC-1 staining (left lower panels). The right panel graphically shows the quantified data. The lower right panel graphically shows the quantified data. Each experiment was performed at least three times, and each slide was captured in at least five different areas. For graphical data, represent mean ± SEM from three independent experiments. Statistical significance was determined using one-way ANOVA followed by Tukey’s post hoc test.

Our results showed that RAW264.7 cells undergoing formation of FC displayed a marked reduction in mitochondrial membrane potential, as evidenced by a decrease in the red-to-green fluorescence ratio, indicating mitochondrial depolarization and dysfunction under lipid-loading conditions. However, after the addition of GGPP to the RAW264.7 cells, the level of MMP was increased during FC differentiation. A pronounced shift toward green fluorescence was observed (*p* < 0.01), consistent with severe mitochondrial depolarization and dysfunction (Figure 3). Indicating that a loss of KLF2 plays a critical role in this process. In contrast, after the addition of GGTI298 to the cells, a significant restoration of MMP (*p* < 0.01) was observed, like the fluorescence patterns approaching those seen in non-differentiated control cells (Figure 3). Our results showed that RAW264.7 cells undergoing formation of FC displayed a marked reduction in mitochondrial membrane potential, as evidenced by a decrease in the red-to-green fluorescence ratio, indicating mitochondrial depolarization and dysfunction under lipid-loading conditions.

### 3.4. Inhibition of KLF2 on the Generation of Intracellular and Mitochondrial ROS

**Figure 4:**
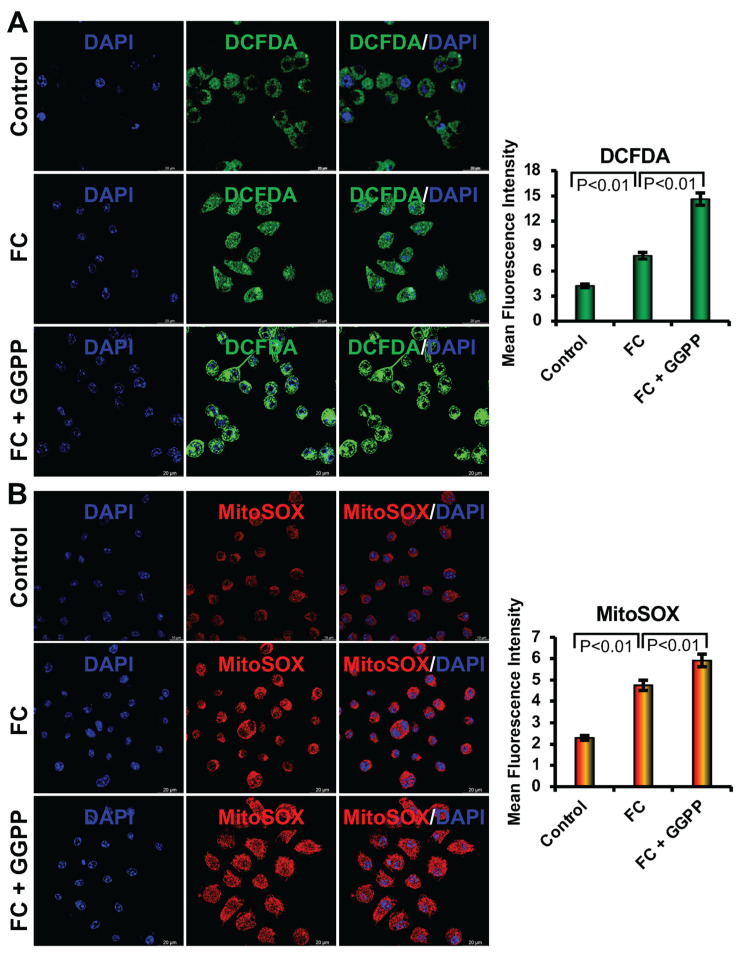
Inhibition of KLF2 increased the intracellular ROS in monocytes, and overexpression of KLF2 reduced it. (**A**). Representative confocal microscopy images were shown after the inhibition of KLF2 (after the addition of GGPP) during foam cell formation with oxidized low-density lipoprotein (ox-LDL) of RAW264.7 cells after DCFDA staining (left upper panels). The upper right panel graphically shows the quantified data. Scale bar = 20 µm. (**B**) Representative confocal microscopy images of MitoSOX staining were shown after the inhibition of KLF2 (after the addition of GGPP) during foam cell formation with ox-LDL of RAW264.7 cells. The lower right panel graphically shows the quantified data. Each experiment was performed at least three times, and each slide was captured in at least five different areas. For graphical data, represent mean ± SEM from three independent experiments. Statistical significance was determined using one-way ANOVA followed by Tukey’s post hoc test.

Oxidative stress is a key driver of FC formation, primarily mediated by the accumulation of reactive oxygen species (ROS) in response to ox-LDL. To evaluate the role of KLF2 in modulating intracellular ROS levels, we performed DCFDA staining followed by confocal microscopy to quantify ROS generation in RAW264.7 cells under various conditions. Our results demonstrated that the addition of the ox-LDL to the RAW264.7 cells significantly elevated intracellular ROS levels compared to the control cells (*p* < 0.01), indicating that lipid oxidation promotes oxidative stress within RAW264.7 cells during FC differentiation. Furthermore, preincubation with GGPP resulted in a further increase in the level of ROS production beyond the induction by ox-LDL alone, suggesting that downregulation of KLF2 exacerbates oxidative stress under atherogenic conditions (Figure 4). Furthermore, the addition of the ox-LDL to the RAW264.7 cells significantly elevated mitochondrial ROS levels compared to the control cells (*p* < 0.01), indicating that lipid oxidation promotes oxidative stress within mitochondria during FC differentiation. Furthermore, after addition of GGPP resulted in a further increase in the level of mitochondrial ROS production beyond the induction by ox-LDL alone, suggesting that downregulation of KLF2 exacerbates oxidative stress at the mitochondrial level also (Figure 4). Collectively, these findings underscore the role of KLF2 in regulating intracellular and mitochondrial redox balance.

### 3.5. Activation of KLF2 on the Generation of Intracellular and Mitochondrial ROS

**Figure 5:**
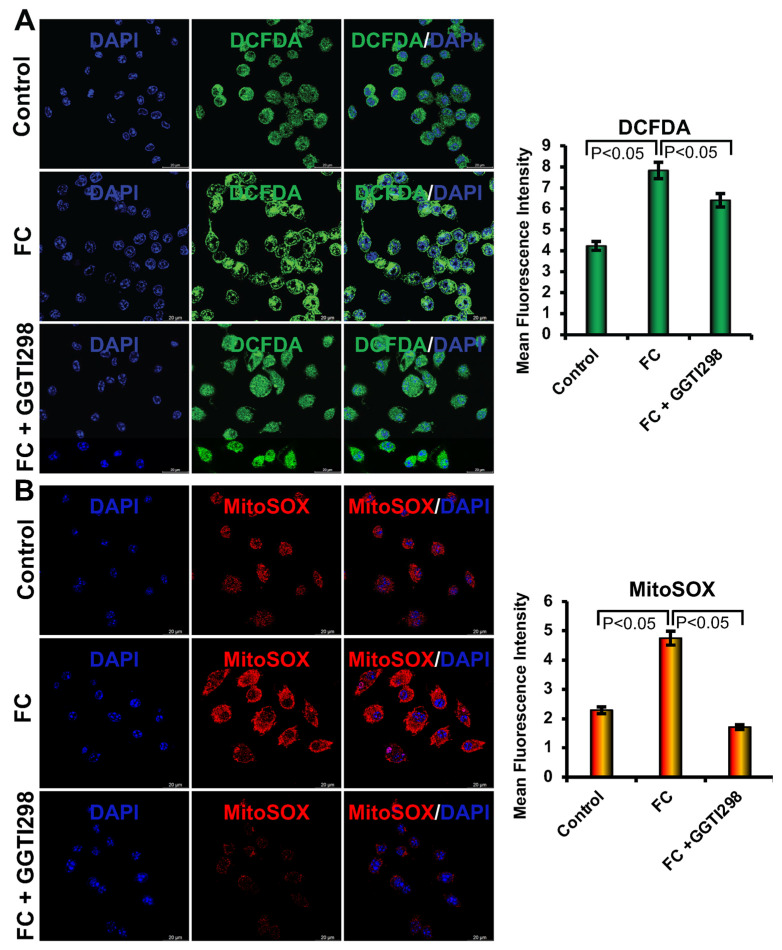
Activation of KLF2 increased the mitochondrial ROS in monocytes, and overexpression of KLF2 reduced it. (**A**). Representative confocal microscopy images were shown after the activation of KLF2 (after the addition of GGTI298) during foam cell formation with oxidized low-density lipoprotein (ox-LDL) of RAW264.7 cells after DCFDA staining (left upper panels). The upper right panel graphically shows the quantified data. (**B**). Representative confocal microscopy images were shown after the overexpression of KLF2 (after the addition of GGTI298) during foam cell formation with oxidized low-density lipoprotein (ox-LDL) of RAW264.7 cells after MitoSOX Red staining (left lower panels). The lower right panel graphically shows the quantified data. Each experiment was performed at least three times, and each slide was captured in at least five different areas. For graphical data, represent mean ± SEM from three independent experiments. Statistical significance was determined using one-way ANOVA followed by Tukey’s post hoc test.

To investigate the role of KLF2 in regulating intracellular and mitochondrial ROS generation during FC differentiation, we conducted DCFDA and MitoSOX Red staining in RAW264.7 cells under various conditions. Briefly, RAW264.7 cells were cultured in FC differentiation media for 6 days with or without the addition of GGTI298. Following differentiation, cells were stained with DCFDA a fluorescent dye that selectively targets intracellular ROS, or MitoSOX Red, a fluorogenic dye that selectively targets mitochondria and fluoresces upon oxidation by superoxide, to assess the levels of mitochondrial ROS. Under FC differentiation conditions, DCFDA staining revealed a substantial decrease in intracellular ROS production, consistent with the reduced oxidative stress. However, when cells were incubated with GGTI298, a downstream metabolite that also activates KLF2 expression, they showed a significant decrease in the level of intracellular ROS (Figure 5). Moreover, the addition of GGTI298 also resulted in a significant reduction in the level of MitoSOX fluorescence intensity, indicating a marked decrease in the generation of mitochondrial ROS. This suggests that KLF2 activation via GGTI298 plays a critical role in suppressing mitochondrial oxidative stress during the FC differentiation process. The reduced mtROS levels observed in GGTI298-treated cells align with the known antioxidant and anti-inflammatory properties of KLF2, reinforcing its protective function in RAW264.7 cells physiology under atherogenic stress (Figure 5).

Together, these results demonstrate that KLF2 is a key regulator of intracellular and mitochondrial redox homeostasis during FC differentiation. By limiting mitochondrial superoxide production, KLF2 may help to protect RAW264.7 cells from oxidative damage, reduce inflammation, and prevent the progression of foam cell formation. These findings further highlight the identification of KLF2 as a target molecule for future development of therapeutics in mitigating mitochondrial oxidative stress associated with cardiovascular diseases.

### 3.6. Effect of Reducing KLF2 on Mitochondrial Activities and Function

**Figure 6:**
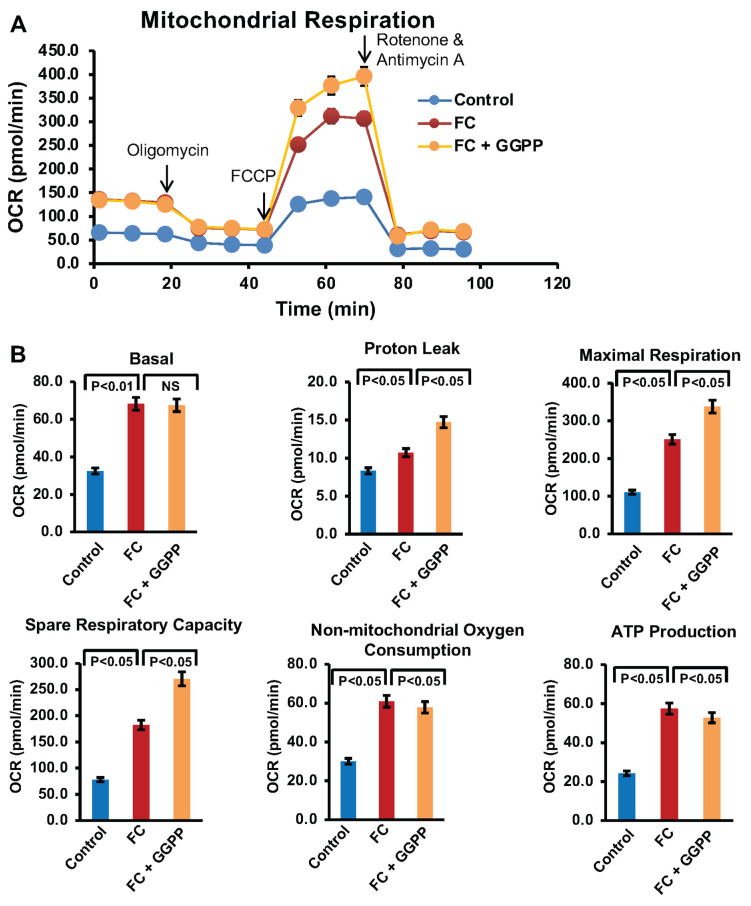
Inhibition of KLF2 further increased the oxidative phosphorylation in RAW264.7 cells during foam cell formation. Seahorse analysis was performed to assess the oxidative phosphorylation in RAW264.7 cells during the foam cell formation with ox-LDL in the presence of GGPP (a potent inhibitor of KLF2), and data were presented in graphical form. (**A**). The line graph represents the raw experimental data. (**B**). Bar graphs show the calculated data for the stated parameters. Data represented as mean ± SEM from three independent experiments. All OCR values were normalized exclusively to cell number. Statistical significance was determined using one-way ANOVA followed by Tukey’s post hoc test.

**Figure 7:**
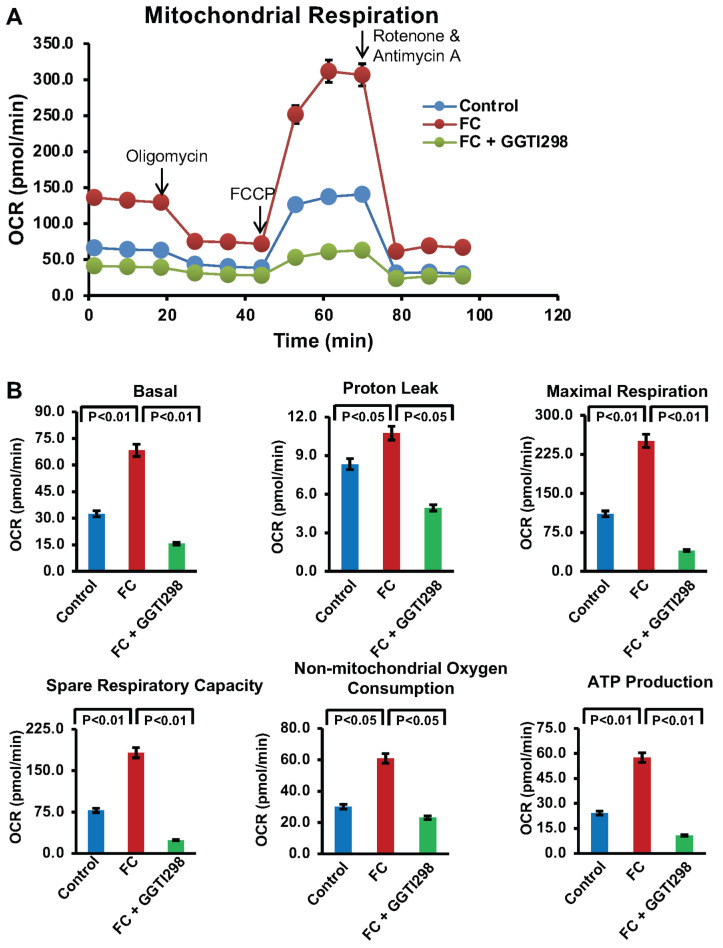
Induction of KLF2 further decreased the oxidative phosphorylation in RAW264.7 cells during foam cell formation. Seahorse analysis was performed to assess the oxidative phosphorylation in RAW264.7 cells during the foam cell formation with ox-LDL in the presence of GGTI298 (a potent activator of KLF2), and data were presented in graphical form. (**A**). The line graph represents the raw experimental data. (**B**). Bar graphs show the calculated data for the stated parameters. All OCR measurements were normalized solely based on cell count. Data represented as mean ±SEM from three independent experiments. Statistical significance was determined using one-way ANOVA followed by Tukey’s post hoc test.

Mitochondria play a central role in regulating cellular energy metabolism and survival by facilitating ATP production through oxidative phosphorylation. During FC formation, RAW264.7 cells undergo significant metabolic reprogramming, making this process highly energy-dependent and reliant on sustained mitochondrial activity [21,22]. To assess the impact of KLF2 on mitochondrial function by measuring oxidative phosphorylation using Seahorse XF Cell Mito Stress analysis, which was performed in live RAW264.7 cells, and analyzed during FC formation. Our results demonstrated that FC formation led to an increase in oxygen consumption rate (OCR), indicative of elevated mitochondrial activity (Figure 6 and Figure 7), which might be associated with foam cell formation.

To confirm the role of KLF2 in this process, we further investigated the reduction in KLF2 on mitochondrial activity. We evaluated mitochondrial respiration after the addition of GGPP during FC formation of RAW264.7 cells. Seahorse’s analysis revealed that the addition of GGPP, which inhibits KLF2 activity, led to a significant increase in most of the OCR parameters (Figure 6A), which might be part of the reasons for hyperactivation in uptaking ox-LDL during FC formation. These included elevated basal respiration, proton leak, maximal respiration, spare respiratory capacity, non-mitochondrial oxygen consumption, and ATP production (Figure 6B). This increase in mitochondrial activity suggests that the downregulation of KLF2 disrupts mitochondrial function and contributes to enhanced oxidative stress during FC formation. Overall, these results highlighted the critical role of KLF2 in regulating mitochondrial dynamics and function in RAW264.7 cells during FC formation.

### 3.7. Effect of Increased KLF2 on Mitochondrial Activity and Function

To further confirm the role of KLF2 in this process, we further investigated the effect of enhanced KLF2 on mitochondrial activity. We evaluated mitochondrial respiration after the addition of GGTI298, a chemical inducer of KLF2, during FC formation of RAW264.7 cells.

Our results demonstrated that FC formation led to an increase in oxygen consumption rate (OCR), indicative of elevated mitochondrial activity (Figure 7A), which might be associated with foam cell formation. However, after the addition of GGTI298, multiple OCR parameters were significantly reduced. Specifically, reductions were observed in basal respiration, proton leak, maximal respiration, spare respiratory capacity, non-mitochondrial oxygen consumption, and ATP production (Figure 7B). These findings confirm that KLF2 overexpression helps maintain mitochondrial homeostasis by suppressing excessive mitochondrial respiration and inhibiting the progression of FC formation.

## 4. Discussion

In this study, we comprehensively investigated the role of Krüppel-like factor 2 (KLF2) in lipid uptake, mitochondrial health, and regulating mitochondrial function during FC formation, with a specific focus on ox-LDL uptake, mitochondrial dynamics, oxidative stress, and energy metabolism. We have reported earlier that KLF2 plays a critical role in maintaining vascular homeostasis, reducing inflammation, and playing an appropriate role in the cell differentiation process [9,10,11,12,16]. However, its role was not investigated elaborately during the FC formation of RAW264.7 cells, as FC formation is the critical, earliest step for the development of plaque formation within the arteries during atherosclerosis pathogenesis. It was shown previously that KLF2 regulates the inflammatory cascade, antioxidant pathways, and mitochondrial functions [15,16]. In RAW264.7 cells exposed to ox-LDL, KLF2 expression is often suppressed; however, whether KLF2 plays a critical role in this process we have delineated its role using RAW264.7 cells and loss-of-function and gain-of-function approaches of KLF2 during FC formation. We found that a significant decrease in the KLF2 expression level occurred after the formation of FC, which is consistent with the previous reports [23]. When we tested the involvement of KLF2 in FC formation, we found that this suppression of KLF2 expression was further intensified upon the addition of GGPP. These findings indicate that the presence of GGPP leads to a substantial loss of the expression of KLF2 during FC formation. The loss of KLF2 might contribute to enhanced FC formation and the development of pathogenesis associated with foam cells. To confirm the role of KLF2 in this process, we have added the GGTI298 to the RAW264.7 cells during the induction of FC. There was a significant induction of KLF2 expression above its basal levels during the FC formation of RAW264.7 cells. These results demonstrate that chemical activation of KLF2 is effective in counteracting the suppressive effects of KLF2 expression during FC formation.

We next investigated the role of KLF2 during the foam cell formation through the uptake of ox-LDL and visualized it using light microscopy. We found that Ox-LDL was uptaken by RAW264.7 cells efficiently to become FCs, which is aligned with the previous reports [24]. However, the lack of KLF2 enhanced the formation of FCs of the monocytes. When we tested the role of KLF2 in this process by adding GGTI298, which caused a substantial reduction in the uptake of ox-LDL by RAW264.7 cells and reduced the formation of FCs. These findings confirmed the regulatory role of KLF2 in the development of FCs from the RAW264.7 cells.

We further observed that when we added ox-LDL to the RAW264.7 cells, there was an increase in both cellular and mitochondrial ROS levels, which are consistent with earlier reports [1,2]. This oxidative stress contributes to depolarization of the mitochondrial membrane and impaired ATP synthesis, as indicated by the decreased red/green JC-1 fluorescence ratio. Activation of KLF2 via GGTI298 reversed this trend, significantly reducing ROS accumulation and restoring mitochondrial membrane potential. This aligns with previous reports showing that KLF2 enhances mitochondrial integrity by promoting antioxidant pathways, such as HO-1 and Nrf2 signaling [16,25].

Moreover, Seahorse XF analysis provided deeper insight into mitochondrial bioenergetics during FC formation. Ox-LDL treatment markedly increased oxygen consumption rate (OCR), particularly basal respiration and ATP-linked respiration, reflecting the mitochondrial overdrive state associated with high energy demand and oxidative stress. Interestingly, KLF2 activation via GGTI298 mitigated this metabolic shift by significantly lowering OCR values across several parameters, thereby suggesting that KLF2 helps restore metabolic balance and reduces electron leakage, a major source of mtROS from Complexes I and III [6,7,8,15]. This regulatory effect likely contributes to the preservation of mitochondrial efficiency and the prevention of foam cell transformation.

In contrast, the suppression of KLF2 using GGPP further exacerbated mitochondrial dysfunction. Not only did GGPP-treated cells show higher lipid accumulation and elevated ROS production, but Seahorse analysis also revealed a dramatic increase in OCR beyond the levels observed in foam cells. This hyperactivation suggests a breakdown in respiratory control, possibly due to unregulated activity at Complex I and III, leading to excessive ROS leakage and mitochondrial damage [1,3]. These results support the view that KLF2 acts as a key modulator of mitochondrial function, potentially inhibiting or regulating Complex I and III activity to limit mtROS production.

Furthermore, the phenocopying of protective effects by rotenone and azoxystrobin, known inhibitors of Complex I and III, respectively, reinforces the hypothesis that KLF2 functionally intersects with these mitochondrial components. Both inhibitors have been shown to reduce VSMC proliferation and ROS generation [5], like KLF2 activation, suggesting a mechanistic link. KLF2 may indirectly modulate the redox activity or conformation of these complexes, thereby attenuating the pro-atherogenic effects of mitochondrial overactivation.

Collectively, our findings support the notion that KLF2 exerts a protective role by modulating mitochondrial reactive oxygen species production and maintaining mitochondrial membrane potential, thus preventing the dysfunctional phenotype associated with foam cells. Importantly, this study reveals that KLF2 influences the activities of mitochondrial Complexes I and III, which are central to ROS generation and energy metabolism in RAW264.7 cells undergoing atherogenic transformation. Overall, our findings position KLF2 as a master regulator of mitochondrial homeostasis during FC formation. By dampening mitochondrial hyperactivity, lowering ROS generation, and stabilizing mitochondrial membrane potential, KLF2 curtails the metabolic and inflammatory cascade that promotes foam cell formation, leading to atherosclerotic plaque formation in the crucial arteries. This dual role in lipid metabolism and mitochondrial regulation makes KLF2 a compelling therapeutic target for early intervention in cardiovascular diseases. A limitation of this study is that KLF2 expression was assessed only at the transcript level. Protein-level validation by Western blotting and/or immunocytochemistry was not performed in the present work. Future studies will be directed toward confirming KLF2 protein expression, which will strengthen the mechanistic link between transcriptional regulation and protein function during foam cell formation. We also acknowledge the limitations of our study. Our findings provide a strong proof-of-concept; future studies should aim to validate these key findings in primary cells, such as murine bone marrow-derived macrophages and human monocyte-derived macrophages, to ensure the physiological relevance of the KLF2-mitochondrial axis in atherogenesis.

## 5. Conclusions

This study highlights the essential role of KLF2 in regulating foam cell formation by maintaining mitochondrial homeostasis, reducing oxidative stress, and limiting lipid uptake in RAW264.7 cells. Loss of KLF2 expression promoted excessive lipid accumulation, mitochondrial depolarization, and elevated ROS generation, all of which contributed to a hyperactive metabolic state that drives foam cell development. In contrast, activation of KLF2 restored mitochondrial function, reduced oxidative stress, and prevented the dysfunctional phenotype characteristic of foam cells.

These findings establish KLF2 as a key modulator of mitochondrial Complex I and III activity, linking lipid metabolism to redox balance during atherogenic stress. By curbing mitochondrial overactivation and stabilizing energy production, KLF2 protects against the early events of atherosclerosis. Taken together, our results underscore the therapeutic potential of targeting KLF2 pathways to slow or prevent cardiovascular disease progression. Future validation in primary murine and human macrophages will be critical for translating these insights into clinically relevant strategies.

## Data Availability

The corresponding author will provide the necessary data supporting the findings of this study upon a reasonable request. The authors are accountable for ensuring the continued availability of the data.

## References

[1] Brand M.D. (2016). Mitochondrial generation of superoxide and hydrogen peroxide as the source of mitochondrial redox signaling. Free Radic. Biol. Med..

[2] Murphy M.P. (2009). How mitochondria produce reactive oxygen species. Biochem. J..

[3] Ballinger S.W. (2005). Mitochondrial dysfunction in cardiovascular disease. Free Radic. Biol. Med..

[4] Tsutsui H., Kinugawa S., Matsushima S. (2009). Mitochondrial oxidative stress and dysfunction in myocardial remodelling. Cardiovasc. Res..

[5] Chouchani E.T., Kazak L., Jedrychowski M.P., Lu G.Z., Erickson B.K., Szpyt J., Pierce K.A., Laznik-Bogoslavski D., Vetrivelan R., Clish C.B. (2016). Mitochondrial ROS regulate thermogenic energy expenditure and sulfenylation of UCP1. Nature.

[6] Xia W., Li Y., Wu M., Yin J., Zhang Y., Chen H., Huang S., Jia Z., Zhang A. (2019). Inhibition of mitochondrial activity ameliorates atherosclerosis in ApoE−/− mice via suppressing vascular smooth cell activation and macrophage foam cell formation. J. Cell. Biochem..

[7] Song Y.J., Zhong C.B., Wang X.B. (2019). Heat shock protein 70: A promising therapeutic target for myocardial ischemia–reperfusion injury. J. Cell. Physiol..

[8] Turrens J.F. (2003). Mitochondrial formation of reactive oxygen species. J. Physiol..

[9] Das H., Kumar A., Lin Z., Patino W.D., Hwang P.M., Feinberg M.W., Majumder P.K., Jain M.K. (2006). Kruppel-like factor 2 (KLF2) regulates proinflammatory activation of monocytes. Proc. Natl. Acad. Sci. USA.

[10] Sen-Banerjee S., Mir S., Lin Z., Hamik A., Atkins G.B., Das H., Banerjee P., Kumar A., Jain M.K. (2005). Kruppel-like factor 2 as a novel mediator of statin effects in endothelial cells. Circulation.

[11] Howlader M.S.I., Prateeksha P., Hansda S., Naidu P., Das M., Barthels D., Das H. (2024). Secretory products of DPSC mitigate inflammatory effects in microglial cells by targeting MAPK pathway. Biomed. Pharmacother..

[12] Das M., Deb M., Laha D., Joseph M., Kanji S., Aggarwal R., Iwenofu O.H., Pompili V.J., Jarjour W., Das H. (2019). Myeloid krüppel-like factor 2 critically regulates K/BxN serum-induced arthritis. Cells.

[13] Laha D., Sarkar J., Maity J., Pramanik A., Howlader M.S.I., Barthels D., Das H. (2022). Polyphenolic compounds inhibit osteoclast differentiation while reducing autophagy through limiting ROS and the mitochondrial membrane potential. Biomolecules.

[14] Greene C.J., Anderson S., Barthels D., Howlader M.S.I., Kanji S., Sarkar J., Das H. (2022). DPSC products accelerate wound healing in diabetic mice through induction of SMAD molecules. Cells.

[15] Zhao X., Xiao Y., Jiang M., Cao Y. (2025). Pharmacological and toxicological roles of Kruppel-like factors (KLFs) in the cardiovascular system: A review. Mol. Biol. Rep..

[16] Maity J., Deb M., Greene C., Das H. (2020). KLF2 regulates dental pulp-derived stem cell differentiation through the induction of mitophagy and altering mitochondrial metabolism. Redox Biol..

[17] Sebti S.M., Hamilton A.D. (2001). Farnesyltransferase and geranylgeranyltransferase I inhibitors as novel agents for cancer and cardiovascular diseases. Farnesyltransferase Inhibitors in Cancer Therapy.

[18] Denizot F., Lang R. (1986). Rapid colorimetric assay for cell growth and survival: Modifications to the tetrazolium dye procedure giving improved sensitivity and reliability. J. Immunol. Methods.

[19] Mosmann T. (1983). Rapid colorimetric assay for cellular growth and survival: Application to proliferation and cytotoxicity assays. J. Immunol. Methods.

[20] Madamanchi N.R., Runge M.S. (2007). Mitochondrial dysfunction in atherosclerosis. Circ. Res..

[21] Vásquez-Trincado C., García-Carvajal I., Pennanen C., Parra V., Hill J.A., Rothermel B.A., Lavandero S. (2016). Mitochondrial dynamics, mitophagy and cardiovascular disease. J. Physiol..

[22] Nunnari J., Suomalainen A. (2012). Mitochondria: In sickness and in health. Cell.

[23] Atkins G.B., Wang Y., Mahabeleshwar G.H., Shi H., Gao H., Kawanami D., Natesan V., Lin Z., Simon D.I., Jain M.K. (2008). Hemizygous deficiency of Kruppel-like factor 2 augments experimental atherosclerosis. Circ. Res..

[24] Attia M.F., Anton N., Wallyn J., Omran Z., Vandamme T.F. (2019). An overview of active and passive targeting strategies to improve the nanocarriers efficiency to tumour sites. J. Pharm. Pharmacol..

[25] Loboda A., Damulewicz M., Pyza E., Jozkowicz A., Dulak J. (2016). Role of Nrf2/HO-1 system in development, oxidative stress response and diseases: An evolutionarily conserved mechanism. Cell. Mol. Life Sci..

